# Rapidly increasing prevalence of overweight and obesity in older Ghanaian adults from 2007-2015: Evidence from WHO-SAGE Waves 1 & 2

**DOI:** 10.1371/journal.pone.0215045

**Published:** 2019-08-19

**Authors:** Stella T. Lartey, Costan G. Magnussen, Lei Si, Godfred O. Boateng, Barbara de Graaff, Richard Berko Biritwum, Nadia Minicuci, Paul Kowal, Leigh Blizzard, Andrew J. Palmer

**Affiliations:** 1 Menzies Institute for Medical Research, University of Tasmania, Hobart, Australia; 2 Research Centre of Applied and Preventive Cardiovascular Medicine, University of Turku, Turku, Finland; 3 The George Institute for Global Health, University of New South Wales, Kensington, NSW, Australia; 4 Department of Nutrition, T.H. Chan School of Public Health, Harvard University, Boston, MA, United States of America; 5 Department of Community Health, University of Ghana, Accra, Ghana; 6 National Research Council, Neuroscience Institute, Padova, Italy; 7 World Health Organization (WHO), Geneva, Switzerland; 8 University of Newcastle Research Centre for Generational Health and Ageing, Newcastle, New South Wales, Australia; 9 Centre for Health Policy, School of Population and Global Health, The University of Melbourne, Melbourne, Victoria, Australia; Dasman Diabetes Institute, KUWAIT

## Abstract

**Background:**

Studies on changes in the prevalence and determinants of obesity in older adults living in sub-Saharan Africa are scarce. We examined recent changes in obesity prevalence and associated factors for older adults in Ghana between 2007/08 and 2014/15.

**Methods:**

Data on adults aged 50 years and older in Ghana were drawn from the WHO SAGE 2007/08 (Wave 1; n = 4158) and 2014/15 (Wave 2; n = 1663). The weighted prevalence of obesity, overweight, normal weight and underweight, and of high central adiposity were compared in 2007/08 and 2014/15. Multinomial and binomial logistic regressions were used to examine whether the determinants of weight status based on objectively measured body mass index and waist circumference changed between the two time periods.

**Results:**

The prevalence of overweight (2007/08 = 19.6%, 95% CI: 18.0–21.4%; 2014/15 = 24.5%, 95% CI: 21.7–27.5%) and obesity (2007/08 = 10.2%, 95% CI: 8.9–11.7%; 2014/15 = 15.0%, 95% CI: 12.6–17.7%) was higher in 2014/15 than 2007/08 and more than half of the population had high central adiposity (2007/08 = 57.7%, 95% CI: 55.4–60.1%; 2014/15 = 66.9%, 95% CI: 63.7–70.0%) in both study periods. While the prevalence of overweight increased in both sexes, obesity prevalence was 16% lower in males and 55% higher in females comparing 2007/08 to 2014/15. Female sex, urban residence, and high household wealth were associated with higher odds of overweight/obesity and high central adiposity. Those aged 70+ years had lower odds of obesity in both study waves. In 2014/15, females who did not meet the recommended physical activity were more likely to be obese.

**Conclusion:**

Over the 7-year period between the surveys, the prevalence of underweight decreased and overweight increased in both sexes, while obesity decreased in males but increased in females. The difference in obesity prevalence may point to differential impacts of past initiatives to reduce overweight and obesity, potential high-risk groups in Ghana, and the need to increase surveillance.

## Introduction

Obesity is a significant global public health challenge because it is a major risk factor for most noncommunicable diseases (NCDs) and independently predicts overall mortality [1–3]. In adults 25 years or older, epidemiological data have been used to establish a causal relationship between high BMI (defined in this study as overweight and obesity) and some chronic diseases such as cardiovascular disease, diabetes mellitus, chronic kidney disease, many cancers and musculoskeletal disorders [3–5]; increased all-cause mortality; and reduced life expectancy [1, 6–9]. For instance, pooled data from four large cohort studies found that the relative risk for each 5 kg/m^2^ higher body mass index (BMI) was 2.32 (2.04–2.63) for diabetes and 1.44 (1.40–1.48) for ischaemic heart disease among those aged 55–64 years [5]. Additionally, high BMI has been associated with poor mental health, reduction in quality-adjusted life years (QALYs), and a high economic burden due to the associated medical and treatment costs [10–16]. In 2015 alone, a total of 603.7 million adults globally were classified to be obese [3]. From 1980 to 2014, obesity has almost doubled among adults in most parts of the world including sub-Saharan Africa [2, 17–19]. Among urban residents in West Africa, the prevalence of obesity has doubled and has consistently increased in both men and women over a period of 15 years from 1992 to 2007 [19, 20].

In Ghana, the increasing prevalence of obesity from 1980 to 2014 among individuals aged 15–49 years has been well-documented [21, 22] and the Ministry of Health found the need to reduce obesity by 2% in the same age group within five years starting from 2008 [23]. However, little is known about the trends in the prevalence of obesity among populations of older adults aged 50 years and above. As a result of improved public health systems, faster fertility transitions, and increased life expectancy, Ghana’s population is rapidly transitioning into an ageing population [24]. This is occurring concurrently with improved nutrition but increased availability of energy-dense foods and increasing sedentary lifestyle that has led to recent increases in obesity prevalence and NCDs [22, 25]. The health and productivity of those in the 50–65 years age range are important as they mentor younger colleagues and form the majority labour force for agricultural productivity that is a key sector for sustainable development and poverty reduction in Ghana [26]. Monitoring trends and identifying factors that relate to obesity provide information that allows interventions to be appropriately and effectively targeted [27, 28] but such data among older adults are lacking in Ghana. Thus, this study aimed to investigate recent changes in the prevalence of obesity among Ghana’s older adult population and identify contributing factors.

## Methods

### Study population

Data from the World Health Organization’s (WHO) 2007/8 (Wave 1) and 2014/15 (Wave 2) Study on global AGEing and adult health (WHO-SAGE) were used. WHO-SAGE is a longitudinal study on the health and well-being of adult populations aged 50 years and older in six countries: China, Ghana, India, Mexico, Russian Federation, and South Africa [29]. In Ghana, trained SAGE teams collected individual-level data from nationally representative households of adults using a stratified, multistage cluster design. The sampling method used in both 2007/08 and 2014/15 was based on the SAGE Wave 0 (2003) in which the primary sampling units were stratified by region and locality (urban/rural) [30, 31]. Weight, height and waist circumference were measured, exempting pregnant women from weight measurements in both surveys [28].

**Fig 1:**
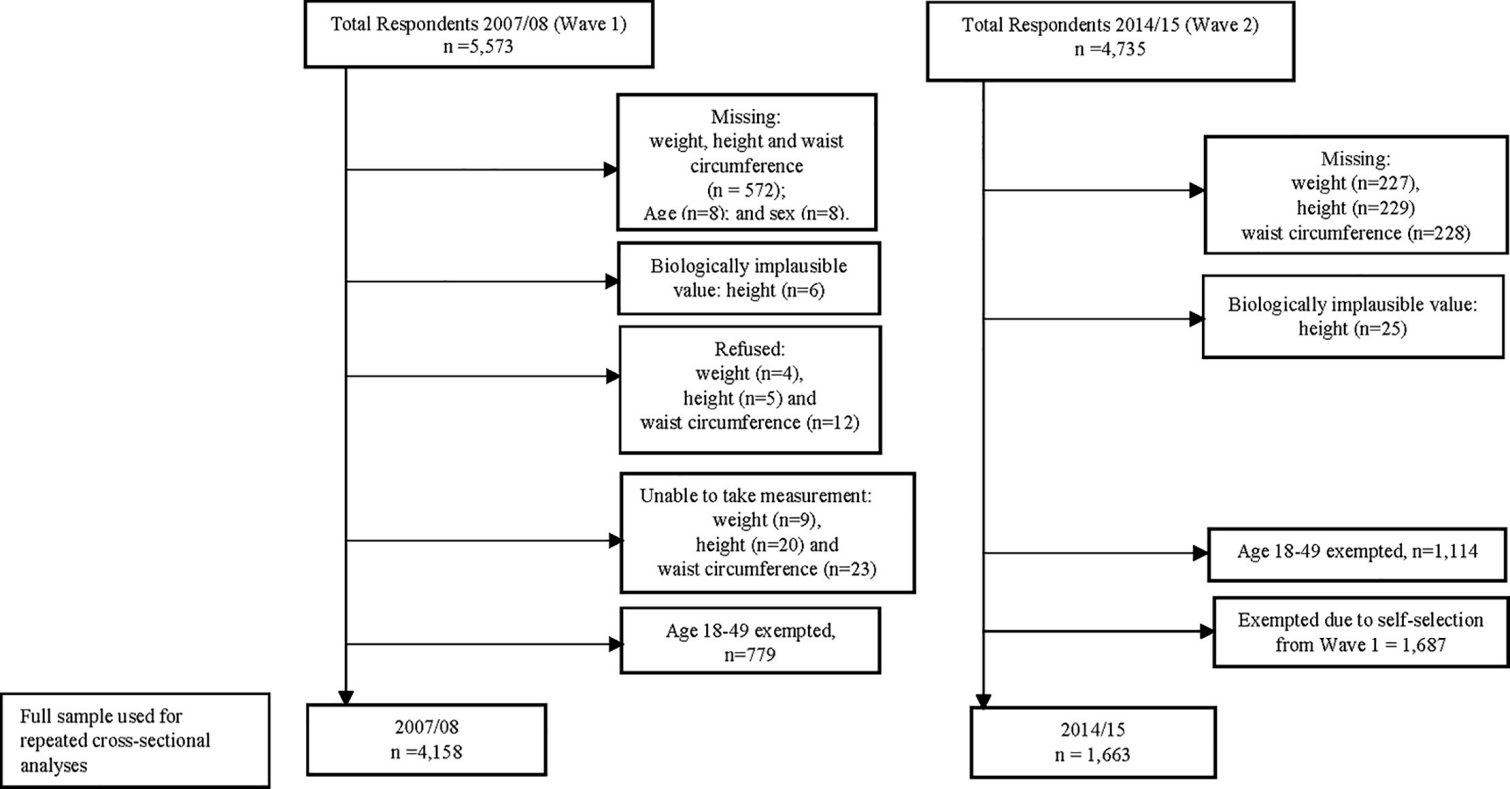
Flowchart showing the analytical sample. Sample extracted from 2007/08 and 2014/15 of the WHO Study on Global AGEing and adult health.

Data collected in 2007/08 had 5,573 and 2014/15 had 4,735 total survey respondents. The final analytical sample at each level of analysis is shown in Fig 1. The final samples for analysis were determined after missing and biologically implausible weight, height and waist circumference measurements were excluded. Biologically implausible values were height <100cm or >250 cm, weight <30.0 kg or >250.0 kg and waist circumference < 25.0 cm or > 220 cm, and were excluded [32, 33]. Additionally, to examine data in a repeated cross-sectional framework and to meet the assumption of independency, all individuals in 2014/15 who participated in 2007/08 were excluded from the analysis [34–36]. A comparison of the “dropped” subjects with the new respondents in terms of BMI and central adiposity was performed (data not shown) and results suggested that the issue of the representativeness should not represent a potential bias. Individuals aged 50+ years with complete responses were 4,158 (2007/08) and 1,663 (2014/15).

### Measures

#### Explanatory factors

The definition of explanatory variables used in this study followed the ecological framework developed by Scott *et al* [37] and adapts the causality continuum model for obesity in sub-Saharan Africa [38]. In their ecological framework, Scott *et al* indicated that factors influencing obesity could be situated in the distant, intermediate and proximate tiers. In the framework, the three tiers are found to interact and overlap with each other, and all influence the health outcome. Distant factors include globalization and urbanization, which affect factors such as lifestyle, food habits, and occupation. The intermediate factors include household and community level characteristics (e.g. household income and cultural perception about weight). Finally, the proximate factors include individual factors that directly affect the health outcome such as a genetic composition of the individual, food habits, and physical activity.

Distant factor: The distant factor included in this study was the location of participants’ residence, i.e. rural or urban residence. Rural or urban residency, the two main types of localities used were defined based on the populations size. A population size less than 5,000 was classified as rural, and larger than 5,000 classified as urban.

Intermediate factors: Household wealth, a proxy for household income representing the economic status of the household [39], was used as an intermediate factor. Even though household wealth is sometimes seen as a poor index for household consumption or expenditure, there has been a consistent lack of data in most low-and middle-income countries to measure the long-term economic status [39–42]. In this study, household wealth was constructed using principal component analysis from 22 items that considered assets and the derived variable was indexed into five quintiles [43].

Proximate factors: These included sex, age, level of education completed and marital status. We also included smoking status, alcohol consumption, fruit and vegetable servings per day and level of physical activity per week. For estimation of age-specific prevalence, age was classified into 10-year intervals. The level of education was grouped into high education (completion of secondary/high school or higher) and low education (highest level of education completed was less than secondary or high school). Marital status was categorized as (1) single, (2) married/cohabiting and (3) divorced/separated/widowed. Respondents' smoking status was coded as (1) "never smoked", (2) "quitter" and (3) "current smoker". Alcohol consumption status was coded as (1) "never", (2) "quitter" and (3) "current drinker". We categorized responses about fruit and vegetable intake according to international standards [44]. Respondents met the recommendation if they consumed ≥5 servings of fruits and/or vegetables per day (equivalent to 400 grams). The level of physical activity was categorised as meeting or not meeting the recommended level of total physical activity per week [45, 46]. Using the Global Physical Activity Questionnaire (GPAQ) instrument that was included in the SAGE, physical activity was estimated by the total metabolic equivalents of task (MET) minutes per week. The analysis guide and STATA syntax was provided by the WHO STEPwise approach to Chronic Disease Risk Factor Surveillance (STEPS) [45]. Meeting the recommended level of total physical activity was defined as engaging in activities including work, during transport and leisure-time for at least 150 minutes of moderate-intensity activity per week or 75 minutes of vigorous-intensity activity per week [46].

### Outcome variables

In WHO SAGE, anthropometric measurements of body weight, height, and waist circumference of respondents were taken by trained interviewers using a weighing scale, stadiometer and Gulick measuring tape following standard protocols [31]. BMI was calculated as a person’s weight in kilograms divided by the square of their height in meters and obesity was defined using cut-offs following the WHO classification. BMI was classified into four categories and weighted prevalence estimated: underweight, BMI<18.50 kg/m^2^; normal/healthy weight, BMI≥18.50<25.00 kg/m^2^; overweight, 25.00<30.00 kg/m^2^; and obese as BMI≥30.00 kg/m^2^ [47]. From the measured waist circumference, high central adiposity was determined using sub-Saharan Africa standards as waist circumference ≥81.2 cm for men, and ≥80.0 cm for women [48].

### Statistical analysis

Accounting for the complex survey design, survey weights were used to estimate age- and sex-specific prevalence of obesity and of high central adiposity. From the cross-sectional datasets, we tested whether the frequency of distribution of the categories of BMI and central adiposity were identical in each wave using the survey Pearson’s chi-squared (*X*^*2*^) test. The absolute and percentage differences in the frequency of each category of BMI and central adiposity between the two waves were calculated for males, females, and both sexes. The categories of BMI and central adiposity were cross-tabulated against the socio-demographic and behavioral factors in S1 and S2 Figs. We fitted survey multinomial and binomial logistic models to estimate odds ratios (OR) and their 95% confidence intervals (CI) of the weight status outcome with sociodemographic and behavioural factors.

The characteristics of the sample are presented in Table 1. Table 2 shows the prevalence, absolute and percentage differences in prevalence. In Tables 3 and 4, the univariable and multivariable regression results are presented in a corresponding manner. BMI categories and central adiposity were the outcome variables whiles the explanatory variables included were age, sex, educational and marital statuses, location of residence, household wealth, smoking and alcohol consumption statuses, fruit and vegetable intake, and total physical activities. Statistical analysis was performed in STATA 15, and a two-tailed *p value<*0.05 was determined as statistically significant.

**Table 1:** Characteristics of older people (50+ years) with complete responses in repeated cross-sectional data from WHO SAGE 2007/08 & 2014/15.

		Males	Females
**2007/08 (n = 4158)**			
n		52.7% (2191)	47.3% (1967)
Mean age, y		64.3 (10.8)	64.2 (10.5)
Mean weight (kg)		62.5 (13.8)	59.7 (15.8)
Mean height (m)		1.66 (8.42)	1.58 (7.42)
Mean waist circumference (cm)		83.3 (12.0)	86.4 (13.5)
^‡^Central Adiposity (WC ≥ optimal cut-point)		50.0% (1090)	66.6% (1317)
Mean BMI (kg/m^2^)		22.8 (5.1)	24.0 (6.0)
BMI Categories	Underweight	15.1% (329)	15.2% (301)
	Normal BMI	59.7% (1302)	49.8% (985)
	Overweight	18.3% (399)	21.1% (417)
	Obese	6.9% (150)	13.9% (275)
**2014/15** (n = 1663)			
n		41.0% (682)	59.0% (981)
Mean age, y		58.2 (8.4)	59.5 (9.1)
Mean weight (kg)		64.6 (11.5)	64.1 (16.3)
Mean height (m)		1.67 (7.78)	1.58 (7.51)
Mean waist circumference (cm)		82.4 (12.9)	89.7 (18.6)
^‡^Central Adiposity (WC ≥ optimal cut-point)		54.4% (370)	75.6% (742)
Mean BMI (kg/m^2^)		23.1 (3.8)	25.8 (6.7)
BMI Categories	Underweight	9.7% (66)	7.9% (77)
	Normal BMI	61.2% (418)	45.4% (446)
	Overweight	23.5% (160)	25.2% (247)
	Obese	5.6% (38)	21.5% (211)

All are weighted estimates. Data are mean (standard deviation) for continuous variables and percentages (sample, n) for categorical variables. BMI indicates body mass index; and WC, waist circumference.

‡ sub-Sahara waist circumference optimal cut-off points for men (WC≥81.2cm) and women (WC≥80.0cm).

**Table 2:** Age- and sex-specific prevalence (95% confidence interval) of body mass index (BMI) categories and high central adiposity (WC ≥ optimal cut-off point) for older people in Ghana using data from 2007/08 (n = 4150) and 2014/15 (n = 1663) of SAGE (All subjects with complete responses used from the repeated cross-sections).

		Underweight	Normal weight	Overweight	Obese		^‡^High Central Adiposity	
		*Pr (95% CI)	Pr (95% CI)	Pr (95% CI)	Pr (95% CI)	*P* Value	Pr (95% CI)	*P* Value
**Total Sample**	2007/08	15.2 (13.6, 16.8)	55.0 (52.8, 57.2)	19.6 (18.0, 21.4)	10.2 (8.9, 11.7)	<0.001	57.7 (55.4, 60.1)	<0.001
** **	2014/15	8.6 (7.1, 10.3)	51.9 (48.8, 55.0)	24.5 (21.7, 27.5)	15.0 (12.6, 17.7)		66.9 (63.7, 70.0)	
** **Absolute difference (95% CI)		-6.6 (-8.6, -4.4)	-3.1 (-6.7, -1.0)	4.9 (1.6, 8.1)	4.8 (2.0, 7.6)		9.2 (5.5, 12.8)	
** **% difference (95% CI)		-43.4 (-52.9, -31.2)	-5.6 (-11.8, 1.0)	25.0 (9.1, 42.7)	47.1 (21.6, 76.9)		15.9 (10.0, 22.0)	
**Males: Age, y -specific prevalence (2007/08**, n = 2180; 2014/15, n = 682)								
50–59	2007/08	11.2 (9.0, 13.9)	60.3 (56.3, 64.1)	21.0 (17.9, 24.5)	7.5 (5.8, 9.8)	0.155	51.0 (46.9, 55.1)	0.252
	2014/15	6.7 (4.3, 10.5)	61.4 (54.9, 67.5)	25.5 (19.5, 32.7)	6.3 (3.6, 10.8)		55.0 (48.6, 61.3)	
60–69	2007/08	14.1 (11.2, 17.7)	62.3 (57.8, 66.5)	16.7 (13.6, 20.4)	6.9 (4.9, 9.5)	0.326	48.7 (43.8, 53.7)	0.323
	2014/15	18.2 (11.5, 27.6)	58.0 (48.1, 67.4)	20.3 (13.3, 29.6)	3.5 (1.5, 8.1)		54.2 (44.3, 63.8)	
≥ 70	2007/08	21.0 (17.0, 25.5)	56.7 (51.9, 61.4)	16.3 (12.8, 20.4)	6.1 (4.0, 9.1)	0.607	49.2 (43.9, 54.7)	0.158
	2014/15	16.0 (9.4, 25.9)	65.5 (54.4, 75.1)	14.8 (8.9, 23.5)	3.8 (1.6, 8.8)		50.9 (41.0, 60.7)	
Total Male sample	2007/08	15.1 (13.3, 17.2)	59.7 (57.0, 62.3)	18.3 (16.2, 20.6)	6.7 (5.6, 8.4)	0.022	49.8 (46.8, 52.8)	0.117
	2014/15	9.7 (7.4, 12.8)	61.2 (56.2, 66.1)	23.5 (18.6, 29.2)	5.6 (3.4, 8.8)		54.4 (49.2, 59.5)	
Absolute difference (95% CI)		-5.4 (-8.6, 2.2)	1.5 (-4.0, 7.1)	5.2 (-0.5, 10.8)	-1.1 (-4.4, 1.7)		4.6 (-1.1, 10.4)	
% difference (95% CI)		-35.8 (-52.2, -13.4)	2.5 (-7.0, 13.3)	28.4 (2.4, 64.1)	-16.4 (-51.0, 33.0)		9.2 (-2.3, 22.1)	
**Females: Age, y -specific prevalence (**2007/08, n = 1398; 2014/15, n = 981)								
50–59	2007/08	9.7 (7.1, 13.2)	45.7 (40.8, 50.6)	24.7 (20.7, 29.1)	20.0 (16.0, 24.6)	0.012	70.6 (66.5, 74.5)	0.014
	2014/15	5.2 (3.7, 7.2)	42.6 (38.2, 47.2)	26.3 (22.7, 30.3)	25.8 (21.6, 30.6)		77.7 (73.3, 81.5)	
60–69	2007/08	12.2 (9.2, 16.1)	53.3 (48.1, 58.5)	22.1 (18.5, 26.2)	12.3 (9.8, 15.5)	0.125	69.6 (64.1, 74.6)	0.389
	2014/15	8.5 (5.3, 13.2)	51.1 (43.6, 58.5)	21.5 (16.6, 27.3)	18.9 (13.0, 26.8)		73.5 (65.7, 80.1)	
≥ 70	2007/08	24.3 (13.0, 19.3)	51.8 (47.4, 56.1)	15.9 (13.0, 19.3)	8.0 (5.8, 10.8)	0.042	59.1 (54.1, 63.9)	0.017
	2014/15	17.9 (12.6, 24.7)	49.1 (41.4, 56.8)	25.5 (18.5, 33.9)	7.6 (4.3, 13.1)		69.8 (62.1, 76.6)	
Total Female Sample	2007/08	15.2 (13.1, 17.5)	49.8 (46.7, 52.8)	21.1 (18.9, 23.5)	13.9 (11.8, 16.4)	<0.001	66.6 (63.6, 69.4)	<0.001
	2014/15	7.9 (7.9, 10.8)	45.4 (41.7, 49.1)	25.2 (22.4, 28.2)	21.5 (18.3, 25.2)		75.6 (71.9, 78.9)	
Absolute difference (95% CI)		-7.3 (-10.0, -4.7)	-4.4 (-8.9, 0.1)	4.1 (0.3, 7.9)	7.6 (3.7, 11.5)		9.0 (4.7, 13.3)	
% difference (95% CI)		-48.0 (-59.4, -34.0)	-8.8 (-16.7, -0.1)	19.4 (2.5, 39.1)	54.7 (26.8, 88.3)		13.5 (7.7, 19.6)	

*Pr (95% CI) means prevalence (95% confidence intervals). All are weighted estimates.

^‡^High central adiposity measured using sub-Sahara high waist circumference cut-off point for men (WC≥81.2cm) and women (WC≥80.0cm)

**Table 3:** Univariable regressions: Factor associated with BMI categories and central adiposity in year WHO-SAGE 2007/08 and 2014/15.

		Year 2007/08			Year 2014/15			Year 2007/08	Year 2014/15
		Underweight	Overweight	Obese	Underweight	Overweight	Obese	High Central Adiposity	High Central Adiposity
		OR (95% CI)	OR (95% CI)	OR (95% CI)	OR (95% CI)	OR (95% CI)	OR (95% CI)	OR (95% CI)	OR (95% CI)
Age Groups, y	50–59	1.00	1.00	1.00	1.00	1.00	1.00	1.00	1.00
	60–69	1.17 (0.88, 1.53)	0.78 (0.62, 0.98) *	0.66 (0.48, 0.89) **	1.95 (1.21, 3.16) **	0.77 (0.55, 1.07)	0.73 (0.45, 1.18)	0.94 (0.79, 1.12)	0.94 (0.70, 1.26)
	≥ 70	2.11 (1.66, 2.69) ***	0.70, (0.56, 0.87) **	0.51 (0.36, 0.74) ***	2.76 (1.70, 4.49) ***	0.79 (0.53, 1.18)	0.35 (0.20, 0.60) ***	0.77 (0.65, 0.93) **	0.84 (0.63, 1.12)
Sex	Male	1.00	1.00	1.00	1.00	1.00	1.00	1.00	1.00
	Female	1.20 (0.97, 1.49)	1.39 (1.13, 1.68) **	2.42 (1.84, 3.20) ***	1.09 (0.75, 1.58)	1.45 (1.05, 2.00) *	4.22 (3.14, 8.70) ***	2.01 (1.72, 2.34) ***	2.59 (1.97, 3.40) ***
Educational Status	Low	1.00	1.00	1.00	1.00	1.00	1.00	1.00	1.00
	High	0.80 (0.62, 1.03)	1.38 (1.10, 1.74)	2.14 (1.61, 2.84)	2.43 (1.42, 4.14) **	0.98 (0.67, 1.43)	0.80 (0.53, 1.21)	1.57 (1.30, 1.91)	0.76 (0.55, 1.06)
Marital status	Single	1.00	1.00	1.00	1.00	1.00	1.00	1.00	1.00
	Married/ cohabiting	0.56 (0.27, 1.17)	1.65 (0.78, 3.49)	1.11 (0.41, 2.98)	1.14 (0.21, 3.31)	1.84 (0.69, 4.85)	0.87 (0.28, 2.73)	1.07 (0.64, 1.80)	0.98 (0.49, 1.96)
	Widow/divorce	0.79 (0.39, 1.60)	1.59 (0.75, 3.38)	1.72 (0.69, 4.31)	1.65 (0.29, 4.45)	1.18 (0.44, 3.18)	0.95 (0.29, 3.08)	1.48 (0.89, 2.46)	0.98 (0.50, 1.93)
Location	Rural	1.00	1.00	1.00	1.00	1.00	1.00	1.00	1.00
	Urban	0.62 (0.47, 0.82) **	2.32 (1.82, 2.97) ***	4.06 (2.56, 7.20) ***	0.47 (0.29, 0.78) **	1.89 (1.36, 2.61) ***	3.47 (2.14, 5.62) ***	2.61 (2.05, 3.32) ***	2.35 (1.73, 3.19) ***
Wealth Index	Lowest	1.00	1.00	1.00	1.00	1.00	1.00	1.00	1.00
	Low	0.84 (0.60, 1.16)	1.73 (1.20, 2.51) **	2.76 (1.29, 5.89) **	1.08 (0.65, 1.79)	1.13 (0.64, 1.99)	1.43 (0.43, 3.73)	1.60 (1.27, 2.02) ***	1.49 (0.99, 2.23)
	Moderate	0.70 (0.52, 0.95) *	2.56 (1.76, 3.72) ***	3.25 (2.04, 6.82) ***	1.43 (0.78, 2.63)	2.00 (1.18, 3.40) *	3.72 (1.98, 6.87) **	2.06 (1.59, 2.66) ***	2.24 (1.44, 3.48) ***
	High	0.70 (0.48, 1.01)	2.66 (1.81, 3.91) ***	3.92 (2.32, 7.44) ***	0.88 (0.46, 1.65)	2.56 (1.53, 4.28) ***	4.26 (2.81, 8.25) ***	2.40 (1.84, 3.12) ***	2.78 (1.75, 4.41) ***
	Higher	0.34 (0.21, 0.54) ***	4.26 (2.33, 7.05) ***	4.94 (2.46, 9.54) ***	0.28 (0.31, 0.60) **	3.27 (1.92, 5.58) ***	5.17 (3.07, 10.10) ***	3.95 (2.80, 6.90) ***	3.84 (2.37, 6.22) ***
Smoking status	Never Smoked	1.00	1.00	1.00	1.00	1.00	1.00	1.00	1.00
	Quitter	0.99 (0.71, 1.40)	0.77 (0.57, 1.05)	0.37 (0.23, 0.60) ***	1.58 (0.50, 3.01)	1.08 (0.40, 2.92)	1.84 (0.31, 4.98)	0.97 (0.76, 1.25)	1.02 (0.36, 2.91)
	Currently smoke	1.78 (1.33, 2.38) ***	0.32 (0.22, 0.47) ***	0.34 (0.17, 0.68) **	1.87 (0.88, 3.97)	0.18 (0.07, 0.48) **	0.18 (0.04, 0.81) *	0.67 (0.55, 0.80) ***	0.23 (0.13, 0.41) ***
Alcohol Consumption Status	Never drunk alcohol	1.00	1.00	1.00	1.00	1.00	1.00	1.00	1.00
	Quitter	0.88 (0.64, 1.21)	0.76 (0.56, 1.03)	1.00 (0.70, 1.44)	0.90 (0.43, 1.91)	0.54 (0.30, 0.98) *	1.57 (0.82, 3.01)	0.59 (0.47, 0.73) ***	1.84 (1.03, 3.27) *
	Currently Drinks	1.18 (0.93, 1.51)	0.57 (0.45, 0.71) ***	0.75 (0.55, 1.02)	0.94 (0.58, 1.51)	0.64 (0.42, 0.97) *	0.69 (0.38, 1.26)	0.37 (0.28, 0.47) ***	0.69 (0.49, 0.96) *
Fruit & Vegetable Intake	Below requirement	1.00	1.00	1.00	1.00	1.00	1.00	1.00	1.00
	Met requirement	0.86 (0.66, 1.12)	1.17 (0.95, 1.42)	1.32 (1.02, 1.72) *	0.57 (0.39, 0.84) **	1.23 (0.91, 1.67)	1.32 (0.91, 1.92)	1.61 (1.32, 1.96) ***	1.27 (0.95, 1.71)
Total Physical Activity	Below requirement	1.00	1.00	1.00	1.00	1.00	1.00	1.00	1.00
	Met requirement	0.85 (0.67, 1.08)	0.88 (0.71, 1.10)	0.64 (0.47, 0.87) **	1.05 (0.72, 1.54)	0.85 (0.62, 1.17)	0.81 (0.55, 1.19)	0.76 (0.63, 0.92) **	0.84 (0.63, 1.11)

*p<0.05

**p<0.01

***p<0.*001;* Fruit & Vegetable Intake (serving per day); Total Physical Activity (Minutes per week); Year is the year for completion of data collection

**Table 4:** Multivariable regressions: Predictors of overweight, obesity and central adiposity in year WHO-SAGE 2007/08 and 2014/15.

		Year 2007/08			Year 2014/15			Year 2007/08	Year 2014/15
		Underweight	Overweight	Obese	Underweight	Overweight	Obese	High Central Adiposity	High Central Adiposity
		OR (95% CI)	OR (95% CI)	OR (95% CI)	OR (95% CI)	OR (95% CI)	OR (95% CI)	OR (95% CI)	OR (95% CI)
Age Groups, y	50–59	1.00	1.00	1.00	1.00	1.00	1.00	1.00	1.00
	60–69	1.17 (0.88, 1.55)	0.79 (0.61, 1.10)	0.67 (0.49, 0.91) *	1.94 (1.19, 3.18) **	0.77 (0.55, 1.09)	0.59 (0.36, 0.97) *	1.01 (0.83, 1.23)	0.93 (0.71, 1.22)
	≥ 70	2.21 (1.73, 2.84) ***	0.70, (0.54, 0.90) **	0.54 (0.36, 0.80) **	2.45 (1.49, 4.04) ***	0.87 (0.57, 1.31)	0.34 (0.18, 0.62) **	0.86 (0.70, 1.07)	0.94 (0.68, 1.29)
Sex	Male	1.00	1.00	1.00	1.00	1.00	1.00	1.00	1.00
	Female	1.23 (0.88, 1.71)	1.47 (1.15, 1.89) **	2.49 (1.74, 3.55) ***	0.81 (0.52, 1.26)	1.70 (1.16, 2.49) **	4.65 (2.65, 9.09) ***	2.03 (1.64, 2.51) ***	3.36 (2.42, 4.68) ***
Educational Status	Low	1.00	1.00	1.00	1.00	1.00	1.00	1.00	1.00
	High	1.34 (1.02, 1.78) *	0.97 (0.73, 1.28)	1.15 (0.83, 1.60)	1.86 (1.00, 3.42)	1.22 (0.80, 1.86)	0.94 (0.61, 1.46)	1.17 (0.93, 1.47)	0.74 (0.49, 1.14)
Marital status	Single	1.00	1.00	1.00	1.00	1.00	1.00	1.00	1.00
	Married/ cohabiting	0.60 (0.29, 1.23)	2.00 (0.92, 4.35)	1.60 (0.64, 3.96)	1.17 (0.17, 2.93)	2.25 (0.81, 4.21)	1.61 (0.63, 4.11)	1.25 (0.69, 2.25)	1.54 (0.69, 3.43)
	Widow/divorce	0.74 (0.37, 1.08)	1.50 (0.68, 3.31)	1.65 (0.69, 3.95)	1.39 (0.19, 3.94)	1.20 (0.44, 3.28)	1.11 (0.43, 2.84)	1.16 (0.67, 2.02)	1.10 (0.46, 2.23)
Location	Rural	1.00	1.00	1.00	1.00	1.00	1.00	1.00	1.00
	Urban	0.79 (0.57, 1.08)	1.44 (1.08, 1.92) *	2.01 (1.41, 2.86) ***	0.53 (0.32, 0.87) *	1.38 (0.96, 1.98)	1.73 (1.03, 2.89) *	1.51 (1.19, 1.92) **	1.91 (1.33, 2.74) **
Wealth Index	Lowest	1.00	1.00	1.00	1.00	1.00	1.00	1.00	1.00
	Low	0.87 (0.62, 1.21)	1.52 (1.05, 2.21) *	2.40 (1.13, 5.13) *	1.12 (0.65, 1.91)	1.13 (0.66, 1.95)	1.53 (0.45, 3.21)	1.31 (1.02, 1.69) *	1.43 (0.93, 2.20)
	Moderate	0.77 (0.51, 1.17)	2.01 (1.33, 3.04) **	3.17 (1.50, 6.68) **	1.91 (1.04, 3.50) *	2.03 (1.16, 3.55) *	3.17 (1.87, 6.31) **	1.56 (1.18, 2.07) **	1.66 (1.02, 2.69) *
	High	0.71 (0.51, 0.97) *	2.15 (1.46, 3.16) ***	3.79 (1.96, 7.93) ***	1.20 (0.68, 2.67)	2.57 (1.50, 4.42) **	3.76 (2.27, 7.28) ***	1.57 (1.16, 2.11) **	2.33 (1.42, 3.81) **
	Higher	0.40 (0.23, 0.68) **	4.42 (2.84, 6.89) ***	4.91 (2.41, 10.17) ***	0.52 (0.22, 1.21)	3.29 (1.89, 5.74) ***	4.67 (2.39, 8.53) ***	3.70 (3.22, 6.85) ***	2.45 (1.39, 4.32) **
Smoking status	Never Smoked	1.00	1.00	1.00	1.00	1.00	1.00	1.00	1.00
	Quitter	1.08 (0.74, 1.57)	0.92 (0.68, 1.26)	0.53 (0.30, 0.92) *	1.68 (0.50, 3.64)	1.73 (0.61, 4.95)	3.48 (0.86, 6.19)	0.77 (0.59, 0.10)	1.06 (0.43, 2.60)
	Currently smoke	1.63 (1.18, 2.25) **	0.54 (0.35, 0.83) **	0.88 (0.46, 1.69)	1.66 (0.69, 4.00)	0.31 (0.11, 0.90) *	0.48 (0.10, 2.23)	0.69 (0.53, 0.90) **	0.44 (0.22, 0.88) *
Alcohol Consumption Status	Never drunk alcohol	1.00	1.00	1.00	1.00	1.00	1.00	1.00	1.00
	Quitter	0.88 (0.63, 1.23)	0.71 (0.51, 0.97)*	0.85 (0.57, 1.29)	0.68 (0.32, 1.43)	0.56 (0.30, 1.06)	1.47 (0.64, 3.38)	0.96 (0.74, 1.24)	2.52 (1.38, 4.59) **
	Currently Drinks	1.19 (0.93, 1.54)	0.64 (0.50, 0.82) ***	0.95 (0.68, 1.31)	0.91 (0.56, 1.49)	0.78 (0.48, 1.27)	1.21 (0.70, 2.08)	0.86 (0.72, 1.03)	1.11 (0.78, 1.58)
Fruit & Vegetable Intake	Below requirement	1.00	1.00	1.00	1.00	1.00	1.00	1.00	1.00
	Met requirement	0.98 (0.75, 1.27)	1.05 (0.85, 1.29)	1.16 (0.90, 1.49)	0.57 (0.37, 0.86) **	1.16 (0.85, 1.60)	1.25 (0.82, 1.90)	1.51 (1.23, 1.84) ***	1.27 (0.92, 1.72)
Total Physical Activity	Below requirement	1.00	1.00	1.00	1.00	1.00	1.00	1.00	1.00
	Met requirement	0.81 (0.64, 1.02)	1.01 (0.80, 1.27)	0.78 (0.57, 1.08)	1.12 (0.73, 1.71)	0.82 (0.59, 1.14)	0.72 (0.49, 1.08)	0.82 (0.67, 0.99) *	0.89 (0.66, 1.20)

*p<0.05

**p<0.01

***p<0.*001;* Fruit & Vegetable Intake (serving per day); Total Physical Activity (Minutes per week); Year is the year for completion of data collection

## Results

In total, 4,158 persons (52% males) provided complete data in 2007/08 whilst 1,663 new persons (41% males) did so in 2014/15. Table 1 presents the estimated frequencies of the characteristics of the study population in 2007/08 and 2014/15. The population in 2014/15 was estimated to be around five years younger and 3.1kg heavier. Particularly for women, the proportion in the overweight/obese and high central adiposity categories were also higher in 2014/15.

The overall age- and sex-specific weighted prevalence of each BMI category and of high central adiposity in the repeated cross-sectional data are shown in Table 2. Relative to 2007/08, the 2014/15 prevalence of overweight (2007/08 = 19.6%; 95% CI: 18.0–21.4%; 2014/15 = 24.5%; 95% CI: 21.7–27.5%) and obesity (2007/08 = 10.2%; 95% CI: 8.9–11.7%; 2014/15 = 15.0%; 95% CI: 12.6–17.7%) was higher. Obesity increased by about 47% while overweight increased by approximately 25% in the population. More than half of the population had high central adiposity in both waves (2007/08 = 57.7%; 95% CI: 55.4–60.1%; 2014/15 = 66.9%; 95% CI: 63.7–70.0%) with about a 16% increase over the 7 to 8-year period. Underweight reduced by about 43% in the population. In 2014/15, despite a decline in the prevalence of obesity for males (2007/08 = 6.7%; 95% CI: 5.6–8.4%; 2014/15 = 5.6%; 95% CI: 3.4–8.8%), the prevalence of overweight was high for males (2007/08 = 18.3%; 95% CI: 16.2–20.6%; 2014/15 = 23.5%; 95% CI: 18.6–29.2%); and the prevalence of both overweight (2007/08 = 21.1%; 95% CI: 18.9–23.5%; 2014/15 = 25.2%; 95% CI: 22.4–28.2%) and obesity (2007/08 = 13.9%; 95% CI: 11.8–16.4%; 2014/15 = 21.5%; 95% CI: 18.3–25.2%) were higher for females. The prevalence of high central adiposity was higher in 2014/15 for both males and females (Table 2).

The distribution of prevalence of the BMI categories by socio-demographic and behavioral factors in 2007/08 and 2014/15 are shown in S1 and S2 Figs. Generally, the prevalence of obesity was high in individuals who resided in urban areas and those from households with high/higher wealth status in both waves. While obesity was high in 2007/08 among those with high education, in 2014/15 it was rather high among both males and females with low education. Obesity was low among females who met the recommended physical activity level in both waves.

Results from the univariable analyses are presented in Table 3. In the multivariable analyses in 2007/08, being female (overweight: OR = 1.47, 95% CI: 1.15–1.89; obesity: OR = 2.49, 95% CI: 1.74–3.55; and high central adiposity: OR = 2.03, 95% CI: 1.64–2.51), living in an urban area (obesity: OR = 2.01, 95% CI: 1.41–2.86; and high central adiposity: OR = 1.51, 95% CI: 1.19–1.92) and those from households with moderate, higher or higher wealth (overweight: OR = 4.42, 95% CI: 2.84–6.89; obesity: OR = 4.91, 95% CI: 2.41–10.17; and high central adiposity: OR = 3.70, 95% CI: 1.22–6.85) were associated with higher odds of overweight, obesity and central adiposity (Table 4). However, being in age group 70+ years (overweight: OR = 0.70, 95% CI: 0.54–0.90; obesity: OR = 0.54, 95% CI: 0.36–0.80), currently smoking status (overweight: OR = 0.54, 95% CI: 0.35–0.83; and high central adiposity: OR = 0.69, 95% CI: 0.53–0.90), and current alcohol drinking (overweight: OR = 0.64, 95% CI: 0.50–0.82) were associated with lower odds of overweight/obesity. Also, being in the 70+ years age group (underweight: OR = 2.21, 95% CI: 1.73–2.84) and currently smoking status (underweight: OR = 1.63, 95% CI: 1.18–2.25) were associated with higher odds of underweight while the odds of underweight was low among those with high/higher household wealth. In 2014/15, most associations found in 2007/08 remained the same with minimal changes in magnitude.

An interaction term between the age categories and sex in a multivariable regression showed that the product of age and sex were not significantly associated with the BMI categories for all age groups in both 2007/08 and 2014/15 except for females between the age of 50–59 years in whom there was a significant association for higher odds of obesity (OR = 2.67; 95% CI: 1.34–5.30) in 2007/08. The same interaction using central adiposity as the outcome also revealed significantly higher odds of high central adiposity among females aged 50–59 years (OR = 1.69; 95% CI: 1.10–2.58) and 60–69 years (OR = 1.54; 95% CI: 1.06–2.25) only in 2007/08. We also examined whether respondents sex modified the association between physical activity and BMI categories and central adiposity. No significant associations were found in 2007/08. However, in 2014/15, not meeting the recommended physical activity level among females was associated with higher odds of obesity (OR = 3.23; 95% CI: 1.13–6.23) and high central adiposity (OR = 2.19; 95% CI: 1.32–3.63).

## Discussion

This study estimated changes in the prevalence and determinants of BMI and central adiposity in the older adult population of Ghana between year and 2007/08 and 2014/15. We found that over the 7 to 8-year period the prevalence of obesity had increased by 47%, overweight by 25%, but underweight reduced by 43%. However, we found heterogeneity by sex with females showing a 55% increase in the prevalence of obesity compared with a 16% reduction among males over the same period. While we provide estimates of the temporal change in BMI categories and high central adiposity for the total population (males and females combined), there are reasons why the sex-stratified estimates should be prioritised. First, as the ratio of males to females reduced between waves, the estimates for the secular changes at the population level are weighted toward females. Second, we observed a sex difference in the temporal change in obesity where males decreased, and females increased from 2007/08 to 2014/15. Being female, living in an urban area and having high household wealth were associated with higher odds of obesity/high central adiposity while those aged 70+ years was associated with lower odds of obesity. Additionally, in 2014/15, not meeting the recommended physical activity among females was associated with higher odds of obesity and central adiposity.

Our findings show that while the prevalence of underweight reduced over the period, overweight, obesity and central adiposity increased over the same period. The increased prevalence of overweight, obesity (in females) and central adiposity could have a negative public health implication as obesity buttresses the increasing burden of NCDs in most low-and middle-income countries. Even though most previous studies in sub-Saharan Africa were cross-sectional studies, most of the studies found a higher prevalence of overweight/obesity in the female population compared to their male counterparts [19, 22, 49, 50]. This phenomenon has been attributed to the body preference of most females [19, 50–53]. However, a recent study of African-Americans suggested that being male with West-African ancestry genes could be protective against obesity specifically, high central adiposity and hence could be a reason for the male/female disparities [54]. Furthermore, high odds of overweight/obesity among African females in many parts of sub-Saharan Africa and elsewhere, have also been largely attributed to general cultural preference in which overweight/obesity is regarded as a source of beauty and a sign of affluence [49, 52]. In Ghana, this could also be attributed to generally low levels of physical activities in the population [49, 55]. This corroborates our finding that low physical activities in females was associated with higher odds of obesity and central adiposity suggesting that promotion of physical activity may support efforts to reduce obesity in females in Ghana as in other population [28, 56].

Overweight and obesity prevalence in both waves was high in females in all age groups with a decline noted after age 59 years. In males, we observed non-significant declines for obesity and increases for high central adiposity across all age groups. The observed difference in trends for obesity and high central adiposity in males was unexpected but could be attributed to the following reasons. First, the differences could reflect where the respective cut-offs for BMI and waist circumference lie on their distributions. As shown in Table 2, whereas about half of the male population had high central adiposity, less than 10% were obese in both waves. Second, this difference could also reaffirm that BMI may not be as sensitive to changes in excess adiposity compared with waist circumference [57].

While obesity prevalence was found to generally increase with age in some developed countries, in most developing countries, it was found to peak around age 50 years, then decline afterward [58]. In this study, a decline in obesity prevalence rates was found after age 59 years in females. Such trends may demand deliberate attention as those aged over 50 years supply the majority of the labour force for agricultural productivity, which has played a major role in sustainable development and poverty reduction in Ghana [26]. Increased obesity prevalence in this age group may lead to higher NCDs and consequently increase overall mortality as well as increasing the medical cost of care [12]. This can further lead to increased absenteeism from work and reduced work productivity, negatively impacting the economy [12, 59].

Urban residency was also associated with higher odds of overweight/obesity and central adiposity in both waves. Residing in an urban area has shown a similar association with obesity in previous studies in Africa and elsewhere [19]. Urban residency is mostly associated with changes in diets and food availability, increased dependence on mechanized transportation, especially in older people coupled with an increasingly sedentary lifestyle and the loss or lack of open and safe places for physical activities [37, 60]. Dietary transition from nutritious foods to the consumption of easily accessible cheap calorie-dense foods as well as longer hours spent on buses and in cars in traffic may have been key drivers of increasing obesity prevalence in urban areas [55, 60, 61], and this is likely to be the same in Ghana. Older people, especially those over the ages 65 years, tend to rely heavily on their children and grandchildren for activities of daily living [62]. These activities include food preparation and timely food supply. These children, who may be busy on the labour market, may be restricted in their ability to prepare home-made foods for them. They may, therefore, resort to purchasing calorie-dense fast-foods. It is also possible that the reliance on others for food sources/preparation amongst those aged over 70 years may contribute to loss of weight, which agrees with our finding that those aged 70+ years had higher odds of underweight.

The finding that high household wealth is associated with higher odds of overweight/obesity and central adiposity in both waves in this population concurs with findings in other low-and middle-income countries [49, 63]. Our finding is the opposite of what is observed in most developed countries where individuals from socioeconomic disadvantaged households tend to be at increased risk of obesity [64, 65]. Household wealth, a proxy for household income, was expected to have a positive impact resulting in good health outcomes for the individuals within a household as it has been in most developed countries [64]. Our findings support Philipson and Posner’s [63] argument that income has a positive impact on weight in less-developed economies; however, as economic development improves, this relationship tends to become negative in the long term. It is suspected that because rich households in LMICs can afford many varieties of foods, increased household wealth could potentially contribute to changes in food preference, increased food consumption and poor choices regarding dietary intake [66].

As part of lifestyle factors, our finding that smoking was associated with lower odds of overweight has been found in previous studies where current smokers were less likely to have increased body weight compared to those who have never smoked before [67]. In 2007/08 quitting smoking was associated with lower odds of obesity however in 2014/15, currently smoking was rather associated with lower odds of overweight which agrees with previous findings in a randomized control trial [68]. As our findings are from repeated cross-sectional studies, we are unable to confer causality. Further studies are necessary to confirm the strength and direction of the association.

This study has several strengths. First, missing from the extant literature is a study that tracks trends in obesity prevalence among older adults in Ghana. We used the most current data to measure prevalence, changes in prevalence and factors associated with overweight/obesity among older adults about whom little is known in sub-Saharan Africa. Second, the prevalence estimates will be useful in establishing and predicting the future economic burden of obesity on health in this population. Third, the inclusion of waist circumference, a marker of central adiposity and a prime marker of cardiometabolic diseases, provides added confirmation of our findings. Finally, the use of a population-based data that is representative of the older adult population, and uses objectively measured rather than self-reported weight, height, and waist circumference to determine obesity in the population are major strengths.

This study had some limitations. First, given that this study uses data from cross-sectional studies, results focused on associations and not causality [49]. Second, the study focuses only on those who were 50 years and above and does not cover the entire population. Therefore, conclusions from this study is limited to the population of 50 years and above. Finally, although the data is representative of the population aged 50 years and over, the analysis omits observations with missing data (13% in 2007/08 and 4.8% in 2014/15) on variables such as weight, height and waist circumference. Hence, there is a chance for selection bias to be introduced that might have affected external validity. However, the analytical sample was large, and the use of the post-stratified persons’ weight supported the analysis.

## Conclusion

We found a decline in underweight among Ghanaian older adults, an increase in overweight among males and females, and an increase in obesity among females only. The Ghana NCDs management strategy 2012–2016 focused on reducing by 2% the overweight and obesity prevalence in females age 15-49years, neglecting those aged 50+ years. However, the exponential increase in the current estimates of overweight and obesity prevalence suggest the need for policy initiatives aimed at reducing overweight and obesity, especially among females aged 50+ years and the importance of advancing surveillance. This study also identifies some factors associated with high obesity prevalence such as being female, living in an urban area and having high household wealth that could inform prevention and intervention programs in improving the health and well-being of older adult populations in sub-Saharan Africa.

## Supporting information

S1 Fig Distribution of underweight, overweight and obesity by socio-demographic factors in the older adult population of Ghana in 2007/08 and 2014/15. (DOCX)

S2 Fig Distribution of underweight, overweight and obesity by behavioral factors in the older adult population of Ghana in 2007/08 and 2014/15. (DOCX)

S1 File Prevalence STROBE statement. (DOCX)
