# Supplementary material for: Rapidly increasing prevalence of overweight and obesity in older Ghanaian adults from 2007-2015: Evidence from WHO-SAGE Waves 1 & 2
Source: PLoS One. 2019 Aug 19;14(8):e0215045. doi: 10.1371/journal.pone.0215045 (PMC6699701; doi:10.1371/journal.pone.0215045)
Supplement: S2 Fig — (DOCX) [file pone.0215045.s002.docx]

| 1. Total physical activity (MET-minutes per week) | 1. Smoking status |
| --- | --- |
|  |  |
| 1. Fruit & Vegetable Intake (meeting recommended number of servings per day) | 1. Alcohol consumption status |
|  |  |
| \| S2 Fig. Distribution of underweight, overweight and obesity by behavioral factors in the older adult population of Ghana in 2007/08 and 2014/15. \| \| --- \| | |
