# Supplementary material for: Rapidly increasing prevalence of overweight and obesity in older Ghanaian adults from 2007-2015: Evidence from WHO-SAGE Waves 1 & 2
Source: PLoS One. 2019 Aug 19;14(8):e0215045. doi: 10.1371/journal.pone.0215045 (PMC6699701; doi:10.1371/journal.pone.0215045)
Supplement: S1 File — (DOCX) [file pone.0215045.s003.docx]

**Title: Rapidly increasing prevalence of overweight and obesity in older Ghanaian adults from 2007-2015: evidence from WHO-SAGE Waves 1 & 2**

**STROBE Statement—**Checklist of items that should be included in reports of cross-sectional studies

|  | **Item No** | **Recommendation** |
| --- | --- | --- |
| **Title and abstract** | 1 | 1. Indicate the study’s design with Title and abstract   **[Within the title page 1 and methods section of the abstract in page 2**] |
|  |  | 1. (b) Provide in the abstract an informative and balanced summary of what was done **[Find in results section of abstract in page 2]** |
| **Introduction** |  |  |
| Background | 2 | Explain the scientific background and rationale for the investigation being reported [**page 4-5**] |
| Objectives | 3 | State specific objectives, including any prespecified hypotheses **[See in page 5]** |
| **Methods** |  |  |
| Study design | 4 | Present key elements of study design early in the paper [**Methods page 6**] |
| Setting | 5 | Describe the setting, locations, and relevant dates, including periods of recruitment, exposure, follow-up, and data collection [**page 6**] |
| Participants | 6 | (a) Give the eligibility criteria, and the sources and methods of selection of participants [**page 6**] |
| Variables | 7 | Clearly define all outcomes, exposures, predictors, potential confounders, and effect modifiers. Give diagnostic criteria, if applicable [**page 6-9**] |
| Data sources/measurement | 8 | For each variable of interest, give sources of data and details of methods of assessment (measurement). Describe comparability of assessment methods if there is more than one group **[page 6-9**] |
| Bias | 9 | Describe any efforts to address potential sources of bias [**page 8 and page 6**] |
| Study size | 10 | Explain how the study size was arrived at **[page 6 and Figure 1**] |
| Quantitative variables | 11 | Explain how quantitative variables were handled in the analyses. If applicable, describe which groupings were chosen and why [**page 7-9**] |
| Statistical methods | 12 | (a) Describe all statistical methods, including those used to control for confounding **[page 9-10]** |
|  |  | (b) Describe any methods used to examine subgroups and interactions [ **page 9-10**] |
|  |  | (c) Explain how missing data were addressed [**page 6 and 22**] |
|  |  | (d) If applicable, describe analytical methods taking account of sampling strategy **[page 9-10**] |
|  |  | (e) Describe any sensitivity analyses [**NA**] |
| **Results** |  |  |
| Participants | 13 | (a) Report numbers of individuals at each stage of study—eg numbers potentially eligible, examined for eligibility, confirmed eligible, included in the study, completing follow-up, and analysed [**page 6, page 11 and Fig 1**] |
|  |  | (b) Give reasons for non-participation at each stage [**page 6**] |
|  |  | (c) Consider use of a flow diagram [**Figure 1**] |
| Descriptive data | 14 | (a) Give characteristics of study participants (eg demographic, clinical, social) and information on exposures and potential confounders [**Please see Results section in page 11-17; Tables 1, 2, 3 and 4; and S1 and S2 Figs**.] |
|  |  | (b) Indicate number of participants with missing data for each variable of interest **[page 6 and 22]** |
| Outcome data | 15 | Report numbers of outcome events or summary measures [**Please see Results section in page 11-17; Tables 1, 2, 3 and 4; and S1 and S2 Figs**] |
| Main results | 16 | (a) Give unadjusted estimates and, if applicable, confounder-adjusted estimates and their precision (eg, 95% confidence interval). Make clear which confounders were adjusted for and why they were included [**Please see** **Tables 3 and 4 in Page 15 and 16**] |
|  |  | (b) Report category boundaries when continuous variables were categorized [**NA**] |
|  |  | (c) If relevant, consider translating estimates of relative risk into absolute risk for a meaningful time period [**NA**] |
| Other analyses | 17 | Report other analyses done—eg analyses of subgroups and interactions, and sensitivity analyses [**Sub-analyses and interactions reported in page 17**] |
| **Discussion** |  |  |
| Key results | 18 | Summarise key results with reference to study objectives [**page 18, paragraph 1**] |
| Limitations | 19 | Discuss limitations of the study, taking into account sources of potential bias or imprecision. Discuss both direction and magnitude of any potential bias [**page 22**] |
| Interpretation | 20 | Give a cautious overall interpretation of results considering objectives, limitations, multiplicity of analyses, results from similar studies, and other relevant evidence [**page 18-21**] |
| Generalisability | 21 | Discuss the generalisability (external validity) of the study results [**page 22**] |
| **Other information** |  |  |
| Funding | 22 | Give the source of funding and the role of the funders for the present study and, if applicable, for the original study on which the present article is based [**page NA**] |
